# Even periconceptional alcohol consumption can have long‐term consequences on heart health in the offspring

**DOI:** 10.1113/EP091190

**Published:** 2023-03-27

**Authors:** Lakshmi Madhavpeddi, Taben M. Hale

**Affiliations:** ^1^ Department of Basic Medical Sciences University of Arizona, College of Medicine – Phoenix Phoenix Arizona USA

**Keywords:** angiotensin, cardiac output, estradiol, ethanol, prenatal programming

1

It is well established that consumption of alcohol during pregnancy negatively impacts fetal development, causing a range of physical and mental impairments (Lunde et al., 2016). As such, women are advised to refrain from alcohol consumption throughout their pregnancy. However, it takes several weeks for a woman to know she is pregnant, and thus she may unknowingly expose her fetus to alcohol early in gestation. The degree to which periconceptional alcohol consumption might impact the cardiovascular health of the offspring was addressed in a recent paper by Dorey et al. (2023). Specifically, the authors evaluated, in rats, fetal cardiac growth, in addition to cardiac function in adult male and female offspring of dams that were exposed to vehicle or ethanol from 4 days before to 4 days after mating. Overall, they report that despite treatment‐related impacts on fetal cardiac growth rates, cardiac function was largely unchanged until 12 months of age, when female offspring of dams exposed to ethanol exhibited a significant reduction in cardiac output. Periconceptional ethanol also resulted in female‐specific increases in in cardiac gene expression of angiotensin II type 1a receptor (*Agtr1a*) and estrogen receptor alpha (*Esr1*) of 19‐month‐old offspring.

Dorey et al. (2023) show that in young adult (5‐ to 7‐month‐old) offspring, cardiac function and acute cardiac functional responses to global ischaemia when measured *ex vivo* are not significantly altered in male or female offspring, regardless of periconceptional ethanol exposure. The finding that cardiac consequences are not revealed until 12 months of age suggests that the increased risk imposed by periconceptional alcohol might be related to an increase in the susceptibility to or degree of pathological remodelling that occurs with ageing. This would be consistent with an increase in left ventricular wall thickness and an increase in *Agtr1a* expression. Angiotensin II type 1a receptor activation in the heart has been shown to promote pathological remodelling and fibrosis, both of which contribute to impaired cardiac function. Indeed, chronic ethanol exposure during pregnancy in rats has been shown to induce cardiac fibrosis in the offspring (Shirpoor & Naderi, 2022). Given that estradiol has been shown to attenuate hypertrophy and fibrosis in the face of increased angiotensin signalling (Shenoy et al., 2009), it is possible that the increase in *Esr1* is a compensatory response to increased local renin–angiotensin system activity. Future studies emanating from these findings might investigate the impact on arterial blood pressure, autonomic regulation of cardiovascular function, and interstitial and perivascular fibrosis in aged offspring. These studies would provide additional insight into the cardiac structure–function relationship in these rats. Identifying the pathophysiology underlying the female‐specific reduction in cardiac output will be important not only for determining the in utero drivers of future cardiac dysfunction attributable to periconceptional ethanol exposure, but also for developing future treatment strategies. Moreover, given the impaired cardiac function in the female offspring, it will be important to evaluate the degree to which these animals are more susceptible to developing heart failure. Specifically, future studies that are designed to evaluate the degree to which periconceptional ethanol exposure confers an increased risk for cardiac dysfunction and pathological remodelling after myocardial infarction or pressure overload would be of significant interest and would provide greater insight into the cardiovascular risk profile in response to early ethanol exposure.

The overall maternal health at the time of conception and throughout pregnancy influences fetal development. Various prenatal exposures have been shown to result in alterations in cardiovascular function in the adult offspring (Lunde et al., 2016; Madhavpeddi et al., 2022). The relative impact on male versus female offspring and the degree of change in cardiovascular function varies with the nature, timing and duration of the exposure. Most studies evaluating the impact of prenatal exposures have focused on later gestational time points or a longer duration of exposure. When women learn that they are pregnant, they typically make dietary and lifestyle changes to ensure optimal fetal development. Gaining a greater understanding of the consequence of exposures during the periconceptional period, before awareness of pregnancy, is of great importance. Although Dorey et al. (2023) showed that periconceptional ethanol exposure did not result in an overt phenotype, the findings suggest that it might induce a female‐specific increase in cardiovascular disease risk or an earlier manifestation of age‐related impairments in cardiac function. This is particularly important in females given the significant increase in heart disease risk that occurs post‐menopause. Gaining a better understanding of these early drivers of cardiovascular disease might present greater opportunities for early interventions to prevent or slow cardiovascular disease progression in individuals at risk.

## AUTHOR CONTRIBUTIONS

Both authors have read and approved the final version of this manuscript and agree to be accountable for all aspects of the work in ensuring that questions related to the accuracy or integrity of any part of the work are appropriately investigated and resolved. All persons designated as authors qualify for authorship, and all those who qualify for authorship are listed.

## CONFLICT OF INTEREST

None declared.
